# Human white matter and knowledge representation

**DOI:** 10.1371/journal.pbio.2005758

**Published:** 2018-04-26

**Authors:** Franco Pestilli

**Affiliations:** Indiana University Bloomington, Department of Psychological and Brain Sciences, Programs in Cognitive Science and Neuroscience, Indiana Network Science Institute, School of Optometry, Departments of Intelligent Systems Engineering and Computer Science, University of Indiana, Bloomington, Indiana, United States of America

## Abstract

Understanding how knowledge is represented in the human brain is a fundamental challenge in neuroscience. To date, most of the work on this topic has focused on knowledge representation in cortical areas and debated whether knowledge is represented in a distributed or localized fashion. Fang and colleagues provide evidence that brain connections and the white matter supporting such connections might play a significant role. The work opens new avenues of investigation, breaking through disciplinary boundaries across network neuroscience, computational neuroscience, cognitive science, and classical lesion studies.

Advancing neuroscientific understanding requires combining experimental data analysis and visualization with theoretical models along three main research axes: brain structure, function, and human behavior. Likely, such understanding will necessitate integration across methodologies, measurement types, and scales, spanning from the micro- (genes’ and proteins’ structure and function) and meso- (neuronal assemblies and local cortical circuitry) to the macroscale (brain cortical folding and white matter tracts) [1–12]. Neuroimaging is the primary method of measurement in living human brains, spanning across all three primary axes and at least two of the measurement scales.

## Evidence for distributed and localized semantic knowledge representation

Critical to understanding brain and behavior is clarifying the principles of human knowledge representation. A plethora of scientific findings from neuroimaging, lesion studies, and computational modeling has sparked debates on the degree to which functions and knowledge are localized to specific regions or distributed across multiple regions of the brain cortex [13–18]

**Fig 1:**
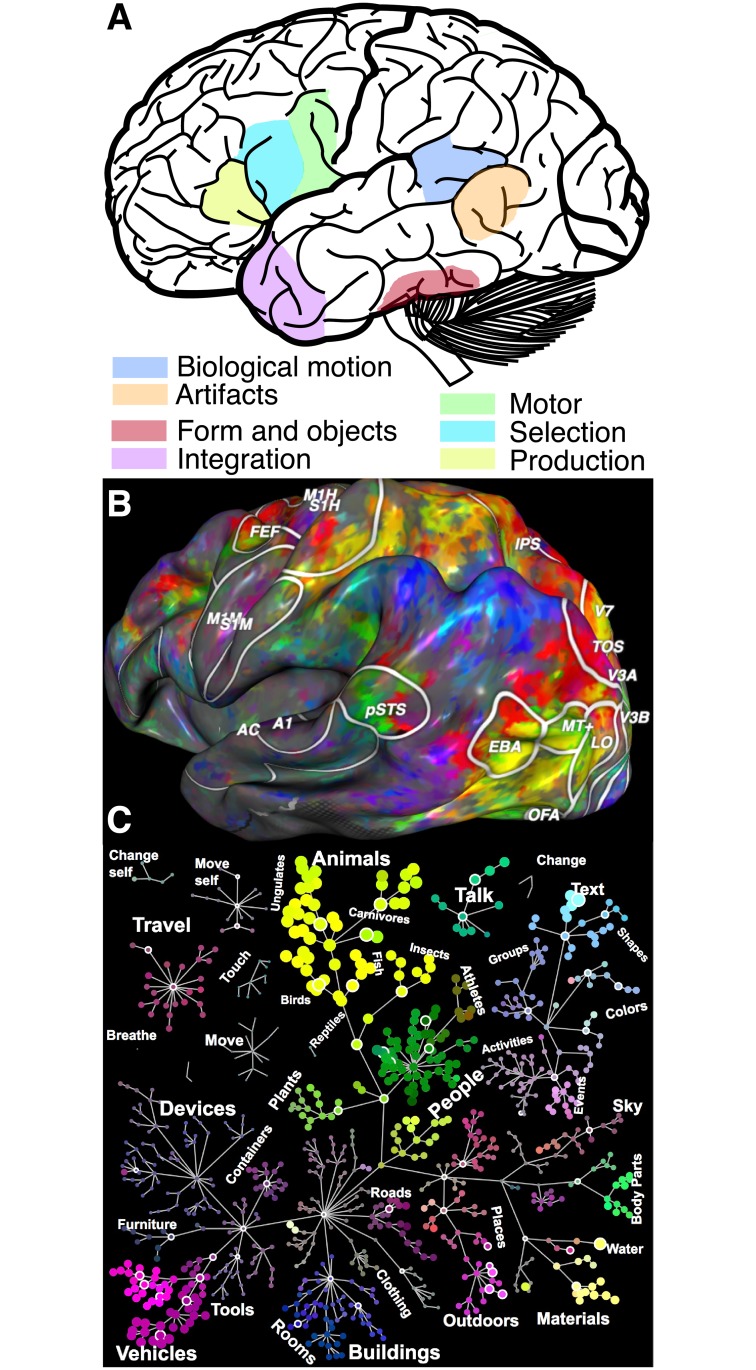
Evidence for distributed or localized information representation in the human brain. (A) Localized representation. The evidence for information representation localization in the human brain is pervasive. This panel shows some of the approximate regions representing different types of information such as information about object form, motion, and object use, as well as information integration. The localized knowledge representation model is perhaps the currently dominant model. It has received converging support from multiple methodologies, suggesting that focalized brain areas represent very specific types of information. (B) Distributed representation. Evidence for distributed brain response to semantic tuning during passive viewing of movie clips [27]. The authors used PCA to map a four-dimensional semantic space and projected each brain location tuning vector onto the second, third, and fourth principal components—visualized as mixtures of red, green, and blue colors. Voxels with similar tuning projected to nearby points in the semantic space are assigned similar colors in this representation and nicely cluster across the cortical mantle. For example, whereas cortical locations colored along the yellow–green axis are more selective to animated objects, body parts and locations colored along the purple–red axis are more selective to spatial locations and movement. (C) Semantic space organization. Objects viewed during movie clips mapped onto the nodes and hierarchical structure of the WordNet lexicon [28]. This visualization allows us to appreciate how semantically neighboring words are represented across spatially neighboring cortical locations as shown in panel A. Figures generated using http://gallantlab.org/index.php/brain-viewer/. *Fig 1A generated combining summary figures reported in* [23] *and* [24]. *Fig 1A brain picture modified from a publicly available file through the Wikimedia Foundation*. PCA, principal component analysis.

Although this commentary is not meant to serve as an exhaustive review, it bears mentioning that on the one hand, since the early work of Broca, Penfield, Meynert, and Wernicke, among others, it has been shown that multiple regions in the human cortex are necessary for or have selective responses to sensory, motor, perceptual, linguistic, or abstract cognitive functions [13,15,18–24]. Indeed, as of today, the dominant model for the representation of semantic knowledge in the human cortex proposes the involvement of multiple spatially localized, functionally specialized but interconnected brain regions (Fig 1A). Several regions of the cortex have been reported to respond preferentially to objects, such as animate or inanimate artifacts, and to motor planning for object-centered actions, among others. Over the years, this model has received substantial evidence from multiple measurement methodologies, both from lesion studies as well as from neuroimaging [18,19,22–26].

On the other hand, perhaps following the legacy of Flourens and Lashley, much work has been reported that is consistent with the existence of distributed representations spanning across multiple cortical regions sharing some degree of functional overlap[14,29,30]. Indeed, human knowledge could, in principle, be distributed dynamically and ubiquitously throughout the cerebral cortex, even though some regions of the cortex might respond preferentially to specific categories of actions or stimuli. In fact, compelling reports have measured semantic knowledge signals distributed across the entire human cortex [16,17,27]. Using functional neuroimaging and advanced computational methods, it has been shown that cortical signals map semantically neighboring concepts to spatially neighboring regions. Furthermore, the semantic representation of the cortical signals can change relative to the allocation of attention during behavioral tasks at hand. These results suggest that the space of semantic knowledge could, in principle, be mapped smoothly and dynamically across the human brain cortex. Fig 1B shows an example of the spatial distribution of brain responses to semantic categories; panel **C** shows the corresponding organization of the semantic knowledge. Color clusters demonstrate continuous but segregated distributions of semantic knowledge. The results can also be taken as an indication that multiple signals could coexist within each brain area. Dynamic signal multiplexing such as the one reported by [16,17,27] has been previously demonstrated in the primate brain [31]. The widespread distribution of semantic signals across the human cortex suggests leveraging the network neuroscience paradigm [9,32,33]. Critical to such a goal is understanding the relation between semantic representation and the pattern of structural brain connections and the white matter tissue within.

## Mapping disconnection syndromes via representational dissimilarity

**Fig 2:**
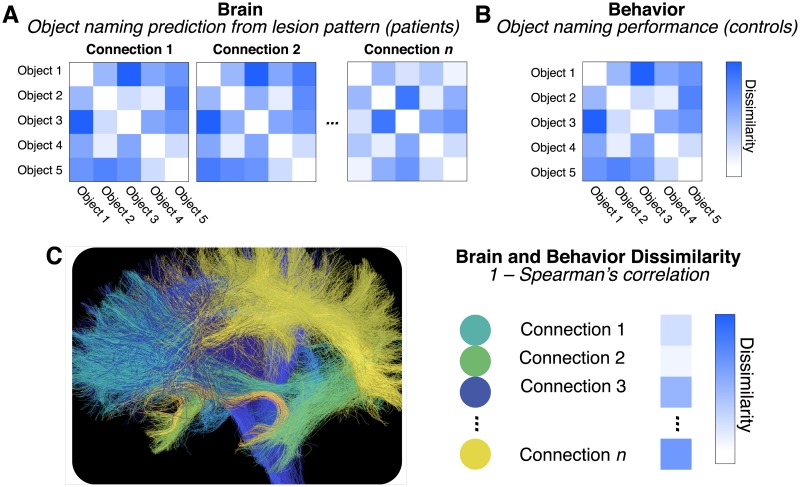
Semantic knowledge representation in distributed brain connections. (A) Brain lesion patterns predict patients’ object-naming performance. Patients’ object-naming performance was predicted using a classification algorithm. The characteristic pattern of brain lesion location and the connections overlapping with the lesions are used in combination with the patients’ naming performance of different objects to predict patients’ naming performance of other objects (e.g., classification performance of object 1 is used to predict objects 2, etc.). (B) Objects’ semantic knowledge structure via behavioral similarity ratings by control subjects. Objects’ similarity was estimated behaviorally in control subjects. Subjects judged objects’ similarity along multiple dimensions (e.g., usage, visual, etc.). (C) Bridging across brain and behavior using representational similarity analysis. The dissimilarity of the objects’ naming prediction from the brain lesion pattern data (A) and the independent objects’ dissimilarity ratings from behavioral responses (B) are correlated. The brain connections overlapping with the lesion pattern were identified. Brain and behavior dissimilarity patterns (1- Spearman’s correlation coefficient) were used to identify connections significant in predicting semantic knowledge. *Brain connection visualization generated using open services and data available at brainlife.io* [38].

The work of Fang and colleagues [34] attempts to relate semantic knowledge—the meaning of words describing objects, things, or actions, such as “dog,” “the sky,” or “travel”—to the structural brain substrates in the human white matter network to demonstrate that semantic representation is not limited to cortical regions but also extends into the connecting white matter pathways [34]. The authors use a clever cocktail of machine learning and computational methods, combining more classical brain lesion data in a group of patients to predict the patients’ object-naming performance (Fig 2A). The pattern of brain lesions was mapped to white matter connections as defined in an atlas [34]. After that, a support vector machine classifier and representational dissimilarity analysis—the inverse of the correlation between brain and behavioral measurements [35–37]—were used to predict patients’ object-naming performance from the brain lesion pattern data in each white matter connection (Fig 2A). In addition, the authors mapped the semantic similarity of a large set of objects in an independent set of control subjects. This mapping allowed them to represent the rich semantic knowledge space of the objects using representational dissimilarity, and such representation was necessary to connect brain connections to behavior (Fig 2). Finally, the two measurement domains for brain and behavior obtained with different data modalities were combined. The authors computed the correlation (Fig 2C, **Brain and behavior**) between the object-naming dissimilarity matrices for each white matter connection collected on the patients’ brain data (Fig 2A, **Brain**), with the behavioral dissimilarity matrix describing the objects’ semantic space collected in the control subjects (Fig 2B, **Behavior**). The procedure allowed the authors to characterize the potential involvement of each connection in the semantic knowledge representation.

**Fig 3:**
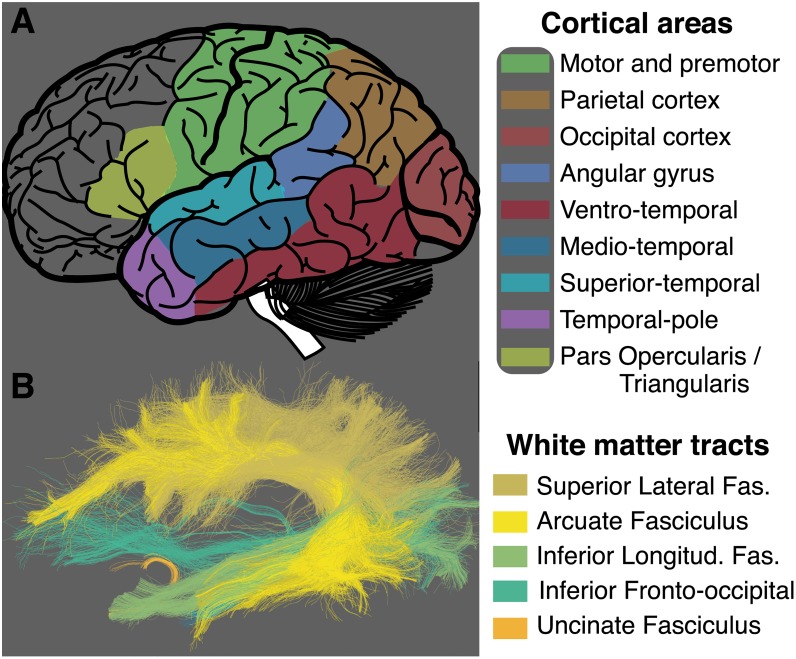
White matter tracts putatively involved in semantic knowledge representation. (A) Some of the cortical areas with connections identified by Fang and colleagues [34] that could potentially play a role in the representation of human semantic knowledge. (B) Major white matter tracts terminating within and between some of the cortical areas identified by Fang and colleagues (A) [34]. The superior lateral fasciculus, the arcuate fasciculus, the inferior longitudinal fasciculus, the inferior fronto-occipital fasciculus, and the uncinate fasciculus all may play a role in coordinating information processing and supporting the computations involved in establishing our sense of knowledge. *Tract visualization generated using open services and data available at brainlife.io* [38]. *Fig 3A brain picture modified from a publicly available file through the Wikimedia Foundation*.

This approach is a clever extension of previous methods using representational dissimilarity analysis on functional neuroimaging data [35] that can be thought of as a modern, computational incarnation of the classical disconnection syndrome analysis [39,40]. The authors identified a series of connections likely to contribute to communicating information relevant for semantic knowledge (see Fig 3 of Fang and colleagues [34]). Several of these connections appear to project to the temporal lobe and map across regions spanning multiple cortical lobes. This is consistent with previous reports on the role of the temporal, parietal, and occipital lobes in language processing, memory, and knowledge representation [22,25,41,42]. Importantly, the large extent of connections and the areas to which they project, speak to the models introduced in Fig 1. Fig 3A shows a map of some of the principal cortical regions whose connections are identified by Fang and colleagues as relevant [34]. This figure was reconstructed for illustration purposes only. To do so, the regions touched by the connections reported by Fang and colleagues were highlighted in different colors [34]. The figure also shows some of the major known human white matter tracts (Fig 3B). These tracts were not reported by Fang and colleagues [34] but were segmented here for illustration purposes using open services and data available at brainlife.io—a modern open cloud services platform for brain analysis—and established methods for human white matter mapping [43–46]. Whereas Fang and colleagues focus on brain connections [34], in the next section, we propose a series of white matter tracts potentially involved in knowledge representation.

## A proposal for white matter tracts potentially involved in semantic knowledge representation

A large body of literature supports the view that long-range brain connections wrapped in myelin sheaths are often bundled together. These bundles of coated axonal projections are generally referred to as white matter tracts or fasciculi [1,5,7,43,47,48]. Fang and colleagues [34] studied brain connections by looking at the cortical terminations of the connections passing through the lesion areas. Their connectivity analyses did not identify, however, which of the known human white matter tracts are involved in the measured behaviors. Yet in principle, this information would be possible and important to obtain because such tracts are highly studied and could provide valuable knowledge for understanding brain disease and even for planning and guiding surgical procedures [5,7,43,49].

A full such analysis of the white matter tracts shared by the reported connections would require substantial additional work and data well beyond that by Fang and colleagues [34]. Nonetheless, I performed some qualitative analyses and attempted to propose some candidate white matter tracts that might be relevant to the findings of Fang and colleagues [34]. Fig 3B shows some of the major white matter tracts whose terminations are known to reside within the cortical regions significantly involved in knowledge representation as reported by Fang and colleagues ([34]; see their Fig 3A and 3B). Given the pattern of connectivity identified, it is likely that at least the inferior fronto-occipital fasciculus, the arcuate fasciculus, the inferior longitudinal fasciculus, the uncinate fasciculus, and the superior lateral fasciculus could be involved in semantic knowledge representation (Fig 3B). Additional white matter tracts could, in principle, also include the vertical occipital fasciculus and the posterior arcuate [50–53]. This is, inevitably, a large number of large white matter tracts. It suggests that a complex behavior, such as knowledge representation, might be implemented by the coordinated activity of multiple brain areas communicating via several of the major brain highways. Given the large-scale involvement of distal cortical areas in human semantic knowledge representation, clarifying the role of the white matter tracts could become critical in understanding the fundamental mechanisms of knowledge representation because of their significance in large-scale brain communication. Importantly, understanding brain communication would inform clinical diagnoses with the potential to clarify the relation between the brain and psychiatric diseases. Fang and colleagues [34] bring a modern, computational approach to one of the classic patient literatures on the disconnection syndromes that is foundational to our understanding of many types of brain disease [39,40,54].

Understanding the human brain is a priority for the scientific community not only because it defines us as species but because psychiatric diseases have profound effects on society. For example, just in Europe, yearly estimates suggest that up to 200 million individuals may be impacted by brain disease, affecting up to 25% of the European population over the course of their lifetime [55–59]. Such impact accounts for up to 800 billion euros in annual costs, representing nearly 38% of the European gross domestic product. These estimates may, unfortunately, be destined to rise because of the steady aging of the European population. The statistics for Europe are just a reflection of a worldwide phenomenon, highlighting the urgent need for better understanding of the human brain. Computational neuroimaging provides some of the most promising approaches to address such profound societal needs [55–59].

We are, in many ways, still in the early stages of understanding the role of human white matter in knowledge representation. More generally, both the structure and functional role of white matter are poorly understood and still reside within the least explored side of brain science. Indeed, only recently have we become aware of the critical role that white matter plays in establishing successful, healthy trajectories across long-term life events such as development and learning and in the aging brain. The properties of white matter tissue within the major white matter tracts have been shown to be associated with human cognitive, perceptual, and emotional abilities and to be critical for a healthy brain [7,8,47,60–62]. Yet much more research will be needed to understand the human white matter.

I like to think about the role of myelin and white matter in the brain as that of a tuning mechanism for a musical instrument. The whole orchestra can be in the theater ready to play a concert, but the performance will fall short unless all instruments are well tuned. The white matter acts as that tuning mechanism that permits the symphony of the brain to attain its most masterful form. Our understanding of brain function will be incomplete without understanding the significance of white matter tissue organization and the pattern of connections it supports [7]. The work of Fang and colleagues [34] helps us move forward by proposing new ways to understand the human brain and cognition. Their results suggest that if knowledge is widely distributed across the cortex, then disruptions to a subset of connections can lead to altered semantic tuning, even though most of the knowledge representations distributed across the cortex would remain intact. Much work will be needed to relate the properties of white matter tissue organization to human knowledge representation, and this avenue of investigation is an exciting and important one.

## References

[1] Goldstone RL, Pestilli F, Börner K. Self-portraits of the brain: cognitive science, data visualization, and communicating brain structure and function. Trends Cogn Sci. 2015;19: 462–474. doi: 10.1016/j.tics.2015.05.012 26187032 10.1016/j.tics.2015.05.012

[2] Sejnowski TJ, Churchland PS, Movshon JA. Putting big data to good use in neuroscience. Nat Neurosci. 2014;17: 1440–1441. doi: 10.1038/nn.3839 25349909 10.1038/nn.3839 PMC4224030

[3] Thompson PM, Stein JL, Medland SE, Hibar DP, Vasquez AA, Renteria ME, et al The ENIGMA Consortium: large-scale collaborative analyses of neuroimaging and genetic data. Brain Imaging Behav. 2014;8: 153–182. doi: 10.1007/s11682-013-9269-5 24399358 10.1007/s11682-013-9269-5 PMC4008818

[4] Martinelli DC, Chew KS, Rohlmann A, Lum MY, Ressl S, Hattar S, et al Expression of C1ql3 in Discrete Neuronal Populations Controls Efferent Synapse Numbers and Diverse Behaviors. Neuron. 2016;91: 1034–1051. doi: 10.1016/j.neuron.2016.07.002 27478018 10.1016/j.neuron.2016.07.002 PMC5017910

[5] Catani M, de Schotten MT. Atlas of Human Brain Connections [Internet]. OUP Oxford; 2012 [Cited April 13 2018]. https://market.android.com/details?id=book-nROILZ9HwEgC

[6] Shadlen MN, Newsome WT. The variable discharge of cortical neurons: implications for connectivity, computation, and information coding. J Neurosci. 1998;18: 3870–3896. Available from: https://www.ncbi.nlm.nih.gov/pubmed/9570816 9570816 10.1523/JNEUROSCI.18-10-03870.1998 PMC6793166

[7] Wandell BA. Clarifying Human White Matter. Annu Rev Neurosci. 2016;39: 103–128. doi: 10.1146/annurev-neuro-070815-013815 27050319 10.1146/annurev-neuro-070815-013815

[8] Fields RD. White matter in learning, cognition and psychiatric disorders. Trends Neurosci. 2008;31: 361–370. doi: 10.1016/j.tins.2008.04.001 18538868 10.1016/j.tins.2008.04.001 PMC2486416

[9] Petersen SE, Sporns O. Brain Networks and Cognitive Architectures. Neuron. Elsevier; 2015;88: 207–219. doi: 10.1016/j.neuron.2015.09.027 26447582 10.1016/j.neuron.2015.09.027 PMC4598639

[10] Südhof TC. Molecular Neuroscience in the 21st Century: A Personal Perspective. Neuron. 2017;96: 536–541. doi: 10.1016/j.neuron.2017.10.005 29096071 10.1016/j.neuron.2017.10.005 PMC5689449

[11] Gold JI, Shadlen MN. The neural basis of decision making. Annu Rev Neurosci. 2007;30: 535–574. doi: 10.1146/annurev.neuro.29.051605.113038 17600525 10.1146/annurev.neuro.29.051605.113038

[12] Amunts K, Lepage C, Borgeat L, Mohlberg H, Dickscheid T, Rousseau M-É, et al BigBrain: an ultrahigh-resolution 3D human brain model. Science. 2013;340: 1472–1475. doi: 10.1126/science.1235381 23788795 10.1126/science.1235381

[13] Kanwisher N. Functional specificity in the human brain: a window into the functional architecture of the mind. Proc Natl Acad Sci U S A. 2010;107: 11163–11170. doi: 10.1073/pnas.1005062107 20484679 10.1073/pnas.1005062107 PMC2895137

[14] Haxby JV, Hoffman EA, Gobbini MI. The distributed human neural system for face perception. Trends Cogn Sci. 2000;4: 223–233. Available from: https://www.ncbi.nlm.nih.gov/pubmed/10827445 10827445 10.1016/s1364-6613(00)01482-0

[15] Thompson-Schill SL, Aguirre GK, D’Esposito M, Farah MJ. A neural basis for category and modality specificity of semantic knowledge. Neuropsychologia. 1999;37: 671–676. Available from: https://www.ncbi.nlm.nih.gov/pubmed/10390028 10390028 10.1016/s0028-3932(98)00126-2

[16] Huth AG, de Heer WA, Griffiths TL, Theunissen FE, Gallant JL. Natural speech reveals the semantic maps that tile human cerebral cortex. Nature. 2016;532: 453–458. doi: 10.1038/nature17637 27121839 10.1038/nature17637 PMC4852309

[17] Huth AG, Nishimoto S, Vu AT, Gallant JL. A continuous semantic space describes the representation of thousands of object and action categories across the human brain. Neuron. 2012;76: 1210–1224. doi: 10.1016/j.neuron.2012.10.014 23259955 10.1016/j.neuron.2012.10.014 PMC3556488

[18] Caramazza A, Shelton JR. Domain-specific knowledge systems in the brain the animate-inanimate distinction. J Cogn Neurosci. 1998;10: 1–34. Available from: https://www.ncbi.nlm.nih.gov/pubmed/9526080 9526080 10.1162/089892998563752

[19] Martin A. The representation of object concepts in the brain. Annu Rev Psychol. 2007;58: 25–45. doi: 10.1146/annurev.psych.57.102904.190143 16968210 10.1146/annurev.psych.57.102904.190143

[20] Mahon BZ, Caramazza A. What drives the organization of object knowledge in the brain? Trends Cogn Sci. 2011;15: 97–103. doi: 10.1016/j.tics.2011.01.004 21317022 10.1016/j.tics.2011.01.004 PMC3056283

[21] Binder JR, Desai RH, Graves WW, Conant LL. Where is the semantic system? A critical review and meta-analysis of 120 functional neuroimaging studies. Cereb Cortex. 2009;19: 2767–2796. doi: 10.1093/cercor/bhp055 19329570 10.1093/cercor/bhp055 PMC2774390

[22] Patterson K, Nestor PJ, Rogers TT. Where do you know what you know? The representation of semantic knowledge in the human brain. Nat Rev Neurosci. Nature Publishing Group; 2007;8: 976 doi: 10.1038/nrn2277 18026167 10.1038/nrn2277

[23] Martin A, Chao LL. Semantic memory and the brain: structure and processes. Curr Opin Neurobiol. 2001;11: 194–201. Available from: https://www.ncbi.nlm.nih.gov/pubmed/11301239 11301239 10.1016/s0959-4388(00)00196-3

[24] Caramazza A, Mahon BZ. The organisation of conceptual knowledge in the brain: The future’s past and some future directions. Cogn Neuropsychol. 2006;23: 13–38. doi: 10.1080/02643290542000021 21049320 10.1080/02643290542000021

[25] Binder JR, Desai RH. The neurobiology of semantic memory. Trends Cogn Sci. 2011;15: 527–536. doi: 10.1016/j.tics.2011.10.001 22001867 10.1016/j.tics.2011.10.001 PMC3350748

[26] Caramazza A, Mahon BZ. The organization of conceptual knowledge: the evidence from category-specific semantic deficits. Trends Cogn Sci. 2003;7: 354–361. doi: 10.1016/s1364-6613(03)00159-1 12907231 10.1016/s1364-6613(03)00159-1

[27] Çukur T, Nishimoto S, Huth AG, Gallant JL. Attention during natural vision warps semantic representation across the human brain. Nat Neurosci. 2013;16: 763–770. doi: 10.1038/nn.3381 23603707 10.1038/nn.3381 PMC3929490

[28] Miller GA. WordNet: A Lexical Database for English. Commun ACM. New York, NY, USA: ACM; 1995;38: 39–41.

[29] Weiner KS, Grill-Spector K. The evolution of face processing networks. Trends Cogn Sci. 2015;19: 240–241. doi: 10.1016/j.tics.2015.03.010 25840651 10.1016/j.tics.2015.03.010 PMC4414913

[30] Rumelhart DE, McClelland JL, Group PR, Others. Parallel distributed processing [Internet]. MIT press Cambridge, MA; 1987 [Cited April 13 2018]. http://www.cs.toronto.edu/~fritz/absps/pdp2.pdf

[31] Huk AC. Multiplexing in the primate motion pathway. Vision Res. 2012;62: 173–180. Available from: https://www.ncbi.nlm.nih.gov/pubmed/22811986 22811986 10.1016/j.visres.2012.04.007 PMC3526112

[32] Mišić B, Sporns O. From regions to connections and networks: new bridges between brain and behavior. Curr Opin Neurobiol. 2016;40: 1–7. doi: 10.1016/j.conb.2016.05.003 27209150 10.1016/j.conb.2016.05.003 PMC5056800

[33] Bassett DS, Sporns O. Network neuroscience. Nat Neurosci. 2017;20: 353–364. doi: 10.1038/nn.4502 28230844 10.1038/nn.4502 PMC5485642

[34] Fang Y, Wang X, Zhong S, Song L, Han Z, Gong G, et al Semantic representation in the white matter pathway. PLoS Biol. 2018;16: e2003993 doi: 10.1371/journal.pbio.2003993 29624578 10.1371/journal.pbio.2003993 PMC5906027

[35] Kriegeskorte N, Mur M, Bandettini P. Representational similarity analysis—connecting the branches of systems neuroscience. Front Syst Neurosci. 2008;2: 4 doi: 10.3389/neuro.06.004.2008 19104670 10.3389/neuro.06.004.2008 PMC2605405

[36] Diedrichsen J, Kriegeskorte N. Representational models: A common framework for understanding encoding, pattern-component, and representational-similarity analysis. PLoS Comput Biol. 2017;13: e1005508 doi: 10.1371/journal.pcbi.1005508 28437426 10.1371/journal.pcbi.1005508 PMC5421820

[37] Kriegeskorte N, Mur M. Inverse MDS: Inferring Dissimilarity Structure from Multiple Item Arrangements. Front Psychol. 2012;3: 245 doi: 10.3389/fpsyg.2012.00245 22848204 10.3389/fpsyg.2012.00245 PMC3404552

[38] Hayashi S, Pestilli F. Reproducible Neuroimaging Via Open Cloud Services: Data Upcycling To Advance Discovery In Network Neuroscience [Internet]. https://brainlife.io. 2017 [Cite April 13 2018].

[39] Catani M, Ffytche DH. The rises and falls of disconnection syndromes. Brain. 2005;128: 2224–2239. doi: 10.1093/brain/awh622 16141282 10.1093/brain/awh622

[40] Geschwind N. Disconnexion Syndromes in Animals and Man Selected Papers on Language and the Brain. Springer, Dordrecht; 1974 pp. 105–236. doi: 10.1007/978-94-010-2093-0_8

[41] Friederici AD, Gierhan SME. The language network. Curr Opin Neurobiol. 2013;23: 250–254. doi: 10.1016/j.conb.2012.10.002 23146876 10.1016/j.conb.2012.10.002

[42] Fedorenko E, Thompson-Schill SL. Reworking the language network. Trends Cogn Sci. 2014;18: 120–126. doi: 10.1016/j.tics.2013.12.006 24440115 10.1016/j.tics.2013.12.006 PMC4091770

[43] Mori S, Wakana S, van Zijl PCM, Nagae-Poetscher LM. MRI Atlas of Human White Matter [Internet]. Elsevier; 2005 [Cited April 13 2018]. https://market.android.com/details?id=book-ltwRYlvFNLIC

[44] Caiafa CF, Pestilli F. Multidimensional encoding of brain connectomes. Sci Rep. 2017;7: 11491 doi: 10.1038/s41598-017-09250-w 28904382 10.1038/s41598-017-09250-w PMC5597641

[45] Pestilli F, Yeatman JD, Rokem A, Kay KN, Wandell BA. Evaluation and statistical inference for human connectomes. Nat Methods. 2014;11: 1058–1063. doi: 10.1038/nmeth.3098 25194848 10.1038/nmeth.3098 PMC4180802

[46] Yeatman JD, Dougherty RF, Myall NJ, Wandell BA, Feldman HM. Tract profiles of white matter properties: automating fiber-tract quantification. PLoS One. 2012;7: e49790 doi: 10.1371/journal.pone.0049790 23166771 10.1371/journal.pone.0049790 PMC3498174

[47] Rokem A, Takemura H, Bock AS, Scherf KS, Behrmann M, Wandell BA, et al The visual white matter: The application of diffusion MRI and fiber tractography to vision scienceRokem et al. J Vis. The Association for Research in Vision and Ophthalmology; 2017;17: 4–4. Available from: http://iovs.arvojournals.org/article.aspx?articleid=2603187 10.1167/17.2.4 PMC5317208 28196374

[48] Schmahmann JD, Pandya DN. Cerebral white matter—historical evolution of facts and notions concerning the organization of the fiber pathways of the brain. J Hist Neurosci. Taylor & Francis; 2007;16: 237–267. Available from: http://www.tandfonline.com/doi/abs/10.1080/09647040500495896 10.1080/09647040500495896 17620190

[49] Catani M, Ffytche DH. The rises and falls of disconnection syndromes. Brain. 2005;128: 2224–2239. doi: 10.1093/brain/awh622 16141282 10.1093/brain/awh622

[50] Takemura H, Rokem A, Winawer J, Yeatman JD, Wandell BA, Pestilli F. A Major Human White Matter Pathway Between Dorsal and Ventral Visual Cortex. Cereb Cortex. 2016;26: 2205–2214. doi: 10.1093/cercor/bhv064 25828567 10.1093/cercor/bhv064 PMC4830295

[51] Yeatman JD, Weiner KS, Pestilli F, Rokem A, Mezer A, Wandell BA. The vertical occipital fasciculus: a century of controversy resolved by in vivo measurements. Proc Natl Acad Sci U S A. 2014;111: E5214–23. doi: 10.1073/pnas.1418503111 25404310 10.1073/pnas.1418503111 PMC4260539

[52] Weiner KS, Yeatman JD, Wandell BA. The posterior arcuate fasciculus and the vertical occipital fasciculus. Cortex. 2017;97: 274–276. doi: 10.1016/j.cortex.2016.03.012 27132243 10.1016/j.cortex.2016.03.012 PMC6760835

[53] Takemura H, Pestilli F, Weiner KS, Keliris GA, Landi SM, Sliwa J, et al Occipital White Matter Tracts in Human and Macaque. Cereb Cortex. 2017;27: 3346–3359. doi: 10.1093/cercor/bhx070 28369290 10.1093/cercor/bhx070 PMC5890896

[54] Charcot JM. Lectures on the Localisation of Cerebral and Spinal Diseases [Internet]. New Sydenham Society; 1883 [Cited April 13 2018]. https://market.android.com/details?id=book-FkYXAQAAMAAJ

[55] Wykes T, Haro JM, Belli SR, Obradors-Tarragó C, Arango C, Ayuso-Mateos JL, et al Mental health research priorities for Europe. Lancet Psychiatry. 2015;2: 1036–1042. doi: 10.1016/S2215-0366(15)00332-6 26404415 10.1016/S2215-0366(15)00332-6

[56] DiLuca M, Olesen J. The cost of brain diseases: a burden or a challenge? Neuron. Elsevier; 2014;82: 1205–1208. Available from: http://www.sciencedirect.com/science/article/pii/S0896627314004887 10.1016/j.neuron.2014.05.044 24945765

[57] Gustavsson A, Svensson M, Jacobi F, Allgulander C, Alonso J, Beghi E, et al Cost of disorders of the brain in Europe 2010. Eur Neuropsychopharmacol. 2011;21: 718–779. doi: 10.1016/j.euroneuro.2011.08.008 21924589 10.1016/j.euroneuro.2011.08.008

[58] Andlin-Sobocki P, Jönsson B, Wittchen H-U, Olesen J. Cost of disorders of the brain in Europe. Eur J Neurol. Wiley Online Library; 2005;12: 1–27. Available from: http://onlinelibrary.wiley.com/doi/10.1111/j.1468-1331.2005.01202.x/full 10.1111/j.1468-1331.2005.01202.x 15877774

[59] Wittchen HU, Jacobi F, Rehm J, Gustavsson A, Svensson M, Jönsson B, et al The size and burden of mental disorders and other disorders of the brain in Europe 2010. Eur Neuropsychopharmacol. 2011;21: 655–679. doi: 10.1016/j.euroneuro.2011.07.018 21896369 10.1016/j.euroneuro.2011.07.018

[60] Thomason ME, Thompson PM. Diffusion imaging, white matter, and psychopathology. Annu Rev Clin Psychol. 2011;7: 63–85. doi: 10.1146/annurev-clinpsy-032210-104507 21219189 10.1146/annurev-clinpsy-032210-104507

[61] Gabrieli JDE. Dyslexia: a new synergy between education and cognitive neuroscience. Science. 2009;325: 280–283. doi: 10.1126/science.1171999 19608907 10.1126/science.1171999

[62] Johansen-Berg H, Baptista CS, Thomas AG. Human structural plasticity at record speed. Neuron. 2012;73: 1058–1060. doi: 10.1016/j.neuron.2012.03.001 22445333 10.1016/j.neuron.2012.03.001 PMC3353540

